# Effect of adhesive coating on calcium, phosphate, and fluoride release from experimental and commercial remineralizing dental restorative materials

**DOI:** 10.1038/s41598-022-14544-9

**Published:** 2022-06-17

**Authors:** Matej Par, Andrea Gubler, Thomas Attin, Zrinka Tarle, Andro Tarle, Katica Prskalo, Tobias T. Tauböck

**Affiliations:** 1 Department of Endodontics and Restorative Dentistry, School of Dental Medicine, University of Zagreb, Gunduliceva 5, Zagreb, Croatia; 2 Department of Conservative and Preventive Dentistry, Center of Dental Medicine, University of Zurich, Plattenstrasse 11, Zurich, Switzerland; 3 Community Health Center Zagreb - Center, Runjaninova 4, Zagreb, Croatia

**Keywords:** Composite resin, Dental materials, Dental biomaterials

## Abstract

This study investigated the potential of adhesive coating for hindering the reactivity of ion-releasing dental restorative materials. Experimental composites were prepared by replacing 10 or 20 wt% of reinforcing fillers with two types of bioactive glass. A glass ionomer, a giomer, and an alkasite were used as representatives of commercial ion-releasing materials. Restorative material specimens were coated with an etch-and-rinse adhesive, 1-step self-etch adhesive, 2-step self-etch adhesive, or left uncoated. The specimens were immersed in a lactic acid solution and ion concentrations were measured in 4 days intervals for 32 days (atomic absorption spectrometry for calcium, UV–Vis spectrometry for phosphate, ion-selective electrode for fluoride, and pH-meter for pH values). The adhesive coating reduced ion release between 0.3 and 307 times, in a significantly material- and adhesive-dependent manner. Fluoride release was most highly impaired, with the reduction of up to 307 times, followed by phosphate and calcium release, which were reduced up to 90 and 45 times, respectively. The effect of different adhesive systems was most pronounced for phosphate release, with the following rankings: uncoated ≥ 2-step self-etch adhesive ≥ 1-step self-etch adhesive ≥ etch-and-rinse adhesive. The differences among adhesives were less pronounced for calcium and fluoride. It was concluded that the resinous adhesive layer can act as a barrier for ion release and diminish the beneficial effects of remineralizing restorative materials.

## Introduction

As secondary caries remains a prevalent shortcoming of permanent dental restorations, the capability of restorative materials to protect dental hard tissues against demineralization through ion release and acid neutralization represents a viable mechanism to extend the service life of restorations^1,2^. Both primary and secondary caries are caused by the imbalance of ions released from and re-precipitated into dental hard tissues under the conditions of decreased pH caused by the metabolic activity of plaque microorganisms^3^. Unlike most conventional, “inert”, restorative materials, some material classes are capable of releasing ions that can be incorporated into dental hard tissues and render them less soluble when exposed to microbial acids^2^. Glass ionomer cements have been traditionally used as remineralizing restorative materials due to their release of fluoride ions that can form fluorapatite in partly demineralized dental hard tissues, thus improving their resistance against acid dissolution. More recent ion-releasing materials include giomers which contain pre-reacted glass ionomer particles in a glass-filled light-curable methacrylate resin, and the so-called “alkasite” which is a dual-curing resin composite functionalized with two types of reactive glass^4^.

In addition to the aforementioned commercial classes of ion-releasing restorative materials, various formulations of experimental composites have been investigated for their potential caries-protective capabilities^5–7^. These composites commonly have a fraction of their reinforcing fillers replaced by reactive fillers, which gradually dissolve in saliva and release remineralizing ions. Among various candidate reactive fillers, bioactive glasses (BGs) have attracted attention due to their capability to simultaneously exert multiple beneficial effects, including the release of remineralizing ions, alkalization of immersion solutions, hydroxyapatite precipitation, and inhibition of bacterial growth^8^. The composition of BGs can be modified and adapted for a particular purpose, for example, by adjusting their reactivity through changes in network connectivity, and incorporating various ions for improved remineralizing (F) or antibacterial effects (Ag, Cu)^9–11^.

Unlike glass ionomers, which are comparatively more hydrophilic and porous, remineralizing resin composites are typically hydrophobic, hence, the dissolution of their reactive fillers is mostly limited to the restoration surface. As resin composites are used in conjunction with adhesive systems, an additional insulating hydrophobic layer covers their surfaces adjacent to dental hard tissues. For remineralizing composites with ion-releasing fillers, this suggests that there are two types of restoration surface with distinctly different reactivities: (I) the outer surface which is directly exposed to water, and (II) the surface bonded to the cavity walls that is covered with an intermediary layer of the adhesive resin. When the bonded interface fails locally due to polymerization shrinkage and mastication forces, the created spaces are filled with saliva and should be protected against secondary caries^12^. Regardless of which part of the adhesive interface fails (dentin/adhesive or adhesive/composite), the layer of adhesive resin remains between the ion-releasing composite and dental hard tissues, presenting a barrier that needs to be surpassed by remineralizing ions.

The effect of a resinous layer on ion release from remineralizing restorative materials has been dominantly investigated for fluoride-releasing materials, namely conventional glass ionomer cements^13,14^, resin-modified glass ionomers^15^, and polyacid-modified composites^16,17^. Within the group of resin composites, the effect has not been extensively investigated and the available data is limited on fluoride release from several outdated composites^13,18^, while the data for more recent materials such as giomers and alkasite is scarce^19^. Within the limitation of having evaluated only fluoride release, all of the aforementioned studies^13–19^ reported that the resinous layer acted as a barrier for ion release, with possible negative implications on the caries-preventive potential of remineralizing restorative materials. Additional evidence is given by an in situ study, which showed that adhesive coating significantly diminished the remineralizing effect of an experimental resin-based calcium phosphate cement^20^. As to our knowledge, no study to date has evaluated the effect of adhesive coatings on ion release from experimental BG-functionalized composites and contemporary commercial materials such as giomers and alkasite.

The present study aimed to investigate the release of calcium (Ca), phosphate (PO_4_), and fluoride (F) ions from experimental BG-containing composites and three commercial restoratives (a glass ionomer, a giomer, and an alkasite) coated with three different adhesive systems. Additionally, pH changes of the immersion medium were measured. The null hypothesis was that adhesive-coated specimens would release similar ion concentrations as uncoated specimens.

## Materials and methods

### Preparation of experimental resin composites

Experimental composites were prepared following the formulation from previous studies^21–23^. The resin system consisted of bisphenol-A-glycidyldimethacrylate (Bis-GMA, Merck, Darmstadt, Germany) and triethylene glycol dimethacrylate (TEGDMA, Merck) in a ratio of 60:40 wt%. Camphorquinone (0.2 wt%; Merck) and ethyl-4-(dimethylamino) benzoate (0.8 wt%; Merck) were added as the photoinitiation system. The resin and photoinitiation system were mixed in a dark bottle using a magnetic stirrer for 48 h.

The fillers for experimental composites consisted of inert glass, silica, and two types of BG (Table 1). The conventional BG 45S5 formulation and the low-Na F-containing BG were prepared via melt-quench route followed by standardized grinding procedures, which ensured comparable particle sizes and theoretical network connectivity (2.1) for both BG types.

**Table 1:** Fillers used in preparation of experimental resin composites.

	Bioactive glass 45S5	Low-sodium fluoride-containing bioactive glass	Inert barium glass	Silica
Particle size (d50)	3 µm	3 µm	1 µm	5–50 nm
Composition (wt%)	45.0% SiO_2_	33.5% SiO_2_	55.0% SiO_2_	> 99.8% SiO_2_
	24.5% CaO	33.0% CaO	25.0% BaO	
	24.5% Na_2_O	10.5% Na_2_O	10.0% Al_2_O_3_	
	6.0% P_2_O_5_	11.0% P_2_O_5_	10.0% B_2_O_3_	
		12.0% CaF_2_		
Silanization (wt%)	None	None	3.2	4–6
Manufacturer	Schott, Mainz, Germany	Schott, Mainz, Germany	Schott, Mainz, Germany	Evonik, Hanau, Germany
Product name/LOT	G018-144/M111473	experimental batch	GM27884/Sil13696	Aerosil R 7200/157,020,635

Experimental composites containing 70 wt% of inorganic fillers were prepared by replacing 0, 10, or 20 wt% of reinforcing fillers with either BG 45S5 or the low-Na F-containing BG (Table 2). The control composite contained only 70 wt% of reinforcing fillers. The inorganic fillers were admixed to the photoactivated resin system using a dual asymmetric centrifugal mixing system (Speed Mixer TM DAC 150 FVZ, Hauschild & Co. KG, Hamm, Germany) at 2000 rpm for 5 min. The mixing was followed by 48 h of dark storage in vacuum to deaerate the composites.

**Table 2:** Composition of experimental resin composites.

Material designation	Filler composition (wt%)			Total filler ratio (wt%)
	Bioactive glass 45S5	Low-sodium fluoride-containing bioactive glass	Reinforcing fillers (inert barium glass : silica = 2:1)	
Control	0	0	70	70
C-10	10	0	60	70
C-20	20	0	50	70
F-10	0	10	60	70
F-20	0	20	50	70

## Reference commercial restorative materials

Three commercial ion-releasing restorative materials were used as references: a giomer (Beautifil II, Shofu, Kyoto, Japan; shade: A2, LOT: 041923), a reinforced glass ionomer (ChemFil Rock, Dentsply Sirona, Konstanz, Germany; shade: A2, LOT: 1903000819), and an “alkasite” material (Cention, Ivoclar Vivadent, Schaan, Liechtenstein; shade: universal, LOT: XL7102).

### Preparation of restorative material specimens

**Figure 1:**
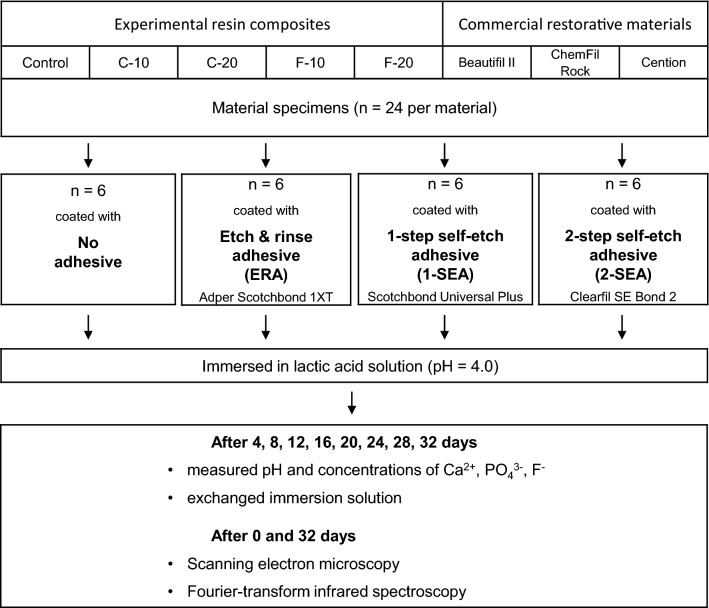
Flowchart of the study design.

The experimental procedure is summarized in Fig. 1. Discoid specimens (diameter = 7 mm, thickness = 2 mm) were prepared by filling polyoxymethylene moulds with uncured materials, covering mould openings with polyethylene terephthalate films, and pressing between thick glass plates to flatten specimen surfaces and extrude excess material. The glass ionomer was allowed to set for 15 min, while the light-curable materials (giomer, alkasite, and experimental composites) were irradiated with 1200 mW/cm^2^ using an LED curing unit (Bluephase PowerCure, Ivoclar Vivadent) for 20 s from each side. After removing the specimen from the mould, flat specimen surfaces were gently polished with ultra-fine (P4000) silicon carbide paper to remove overhangs and obtain a uniform specimen polish. The specimens (n = 24 per material) were separated into a control group (no adhesive coating) and three experimental groups coated with one of the following adhesive systems: an etch-and-rinse adhesive (ERA) Adper Scotchbond 1XT (3 M, St Paul, MN, USA, LOT: NC68002), a universal, i.e. one-bottle self-etch adhesive (1-SEA) Scotchbond Universal Plus (3 M, LOT: 90936A), and a two-bottle self-etch adhesive (2-SEA) Clearfil SE Bond 2 (Kuraray, Tokyo, Japan, LOT: 8J0150 (primer), C30230 (bond)). The adhesive systems were applied according to their respective manufacturer instructions and light-cured using Bluephase PowerCure (Ivoclar Vivadent) with 1200 mW/cm^2^ for 10 s. For the 2-SEA, two-step application was performed in inverse order compared to the clinical application of the primer and the bond component, i.e., the bond component was applied first onto the specimen surface, followed by air-thinning and applying the primer component. The primer component was subsequently air-thinned and light-cured for 10 s with 1200 mW/cm^2^. This was done to ensure that the components were layered in the same order as in clinical application, i.e., that the bond component is the first layer on the restoration surface, which is overlayered with the primer component. Coating the outer specimen surfaces with an adhesive was chosen as a feasible approach for in vitro investigation of ion release through an adhesive layer, as described in several previous studies^13,15,16,18,20^.

### Immersion of specimens in lactic acid solution

After setting, each restorative material specimen was immersed individually in 5 mL of a lactic acid solution of pH = 4.0 in closed Eppendorf vials at room temperature (23 ± 1 °C). The immersion solution was agitated using a horizontal shaker at 30 revolutions per minute. Ion concentrations (Ca, PO_4_, and F) and pH were measured after 4, 8, 12, 16, 20, 24, 28, and 32 days. At each time point, a fresh 5 mL of the lactic acid solution was added to the vial. The immersion conditions and measurement times were selected following previous studies^21–23^. Ion release in aqueous lactic acid solution has been commonly used in investigations of ion-releasing restorative materials, including contemporary resin composites, glass ionomers, compomers, resin-modified glass ionomers, as well as various experimental ion-releasing materials^24–28^.

### Measurements of ion concentrations and pH

The experimental protocol for measuring Ca, PO_4_, and F concentrations is described in detail in a previous study^23^. Briefly, Ca concentrations were measured using an atomic absorption spectrometer (Contra 300, Analytik Jena, Jena, Germany) at a wavelength of 422.67 nm and the following instrument parameters: flame type: C_2_H_2_–N_2_O, C_2_H_2_-air flow rate: 80 L/h, C_2_H_2_–N_2_O: flow rate 215 L/h, and burner height: 5 mm. PO_4_ concentrations were evaluated by means of the malachite green method^29^ and measuring absorbance at 750 nm using a microplate reader (SpectraMax M2e, Molecular Devices, San Jose, CA, USA). F concentrations were measured using a pH/multimeter (Combilab 1254, Systag, Rüschlikon, Switzerland) with a fluoride electrode (Combination Fluoride Electrode 9609BNWP, Thermo Fisher Scientific, Chelmsford, MA, USA). One millilitre of immersion media was mixed with 1 ml of total ionic strength adjustment buffer and F concentrations were measured after 5 min. pH was measured using a pH meter (780 pH Meter, Metrohm, Herisau, Switzerland) equipped with a micro-electrode (Biotrode, Metrohm, Herisau, Switzerland).

### Scanning electron microscopy

Scanning electron microscopic images were taken before and after the 32 days lactic acid immersion. Two specimens per experimental group were rinsed with deionized water, dried at room temperature for 14 days, and sputter-coated with 5 nm of gold. A scanning electron microscope (SEM; Zeiss Gemini 450, Carl Zeiss, Oberkochen, Germany) was used at 10 kV, working distance of 12.5–13.5 mm, vacuum set to 200 Pa, and 2000–50000 × magnification.

### Fourier-transform infrared spectroscopy

Before and after the 32 days lactic acid immersion, two specimens per experimental group were inspected using Fourier-transform infrared (FTIR) spectroscopy. Specimen surfaces were pressed in contact with the diamond attenuated total reflectance (ATR) accessory of the Nicolet iS50 FTIR spectrometer (Thermo Fisher Scientific, Waltham, MA, USA). Thirty-two scans per spectrum were recorded in absorbance mode using a mercury-cadmium-telluride detector. The spectral range was 3500–400 cm^−1^ with a resolution of 4 cm^−1^. Hydroxyapatite was identified using spectral bands at 560 and 600 cm^−1^ assigned to PO_4_ bending^9^. Spectral bands specific for dental restorative materials and adhesive systems were assigned according to references^30,31^.

### Statistical analysis

The data were inspected for normality using Shapiro Wilk’s test and normal Q-Q plots. Two-way ANOVA was performed to evaluate partial eta-squared values for the factors “material” and “adhesive”. This analysis of effect size was performed overall (for all materials together) and separately within each material group, i.e., the group of five experimental composites and the group of three commercial materials.

For each material, one-way ANOVA with Tukey post-hoc adjustment was used to compare cumulative 32 days ion concentrations and pH values among different adhesive coatings. The overall level of significance for all comparisons was 0.05. The statistical analysis was performed using SPSS (version 25; IBM, Armonk, NY, USA).

## Results

Partial eta-squared values indicating relative effect sizes of the material and adhesive type are shown in Table 3. Considering all materials together, the material and adhesive type had a higher effect size (partial eta-squared in parentheses) on the concentrations of Ca and F (0.783–0.946) compared to PO_4_ concentrations and pH (0.395–0.550). The same relative strengths of the effects of material and adhesive type being more influential for Ca and F concentrations than PO_4_ concentrations and pH were observed when the experimental composites were considered separately. This pattern was not observed for the commercial materials, which had the concentrations of all three ion types (Ca, PO_4_, and F) similarly affected by the factors material and adhesive. In the comparison of commercial vs. experimental materials, the adhesive type had a more pronounced effect on all ion concentrations and pH in the group of commercial materials.

**Table 3:** Partial eta-squared values as a measure of the effect size for factors “material” and “adhesive”.

	Experimental materials	Commercial materials	All materials
**Material**			
Calcium	0.634	0.902	0.783
Phosphate	0.133	0.868	0.550
Fluoride	0.961	0.755	0.933
pH	0.421	0.508	0.431
**Adhesive**			
Calcium	0.779	0.927	0.846
Phosphate	0.359	0.836	0.530
Fluoride	0.967	0.975	0.946
pH	0.196	0.951	0.395

**Figure 2:**
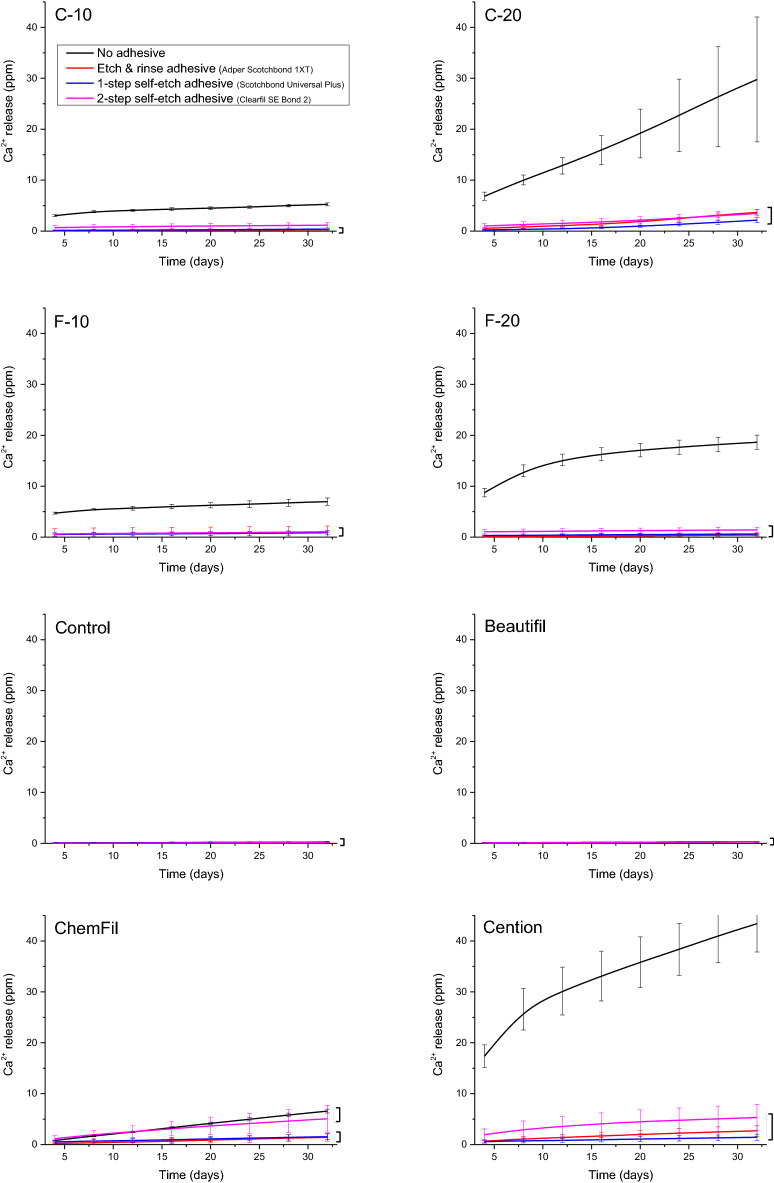
Cumulative concentrations of calcium ions measured in lactic acid solution over 32 days. Square brackets indicate statistically similar values at the end of the observation period.

Considering the Ca-releasing materials (all materials except Control and Beautifil), significantly lower Ca concentrations were measured for adhesive-coated specimens compared to uncoated specimens (Fig. 2). Statistically significant differences among adhesive types were identified only for C-10 and ChemFil in which 2-SEA showed significantly higher Ca concentrations than 1-SEA and ERA.

**Figure 3:**
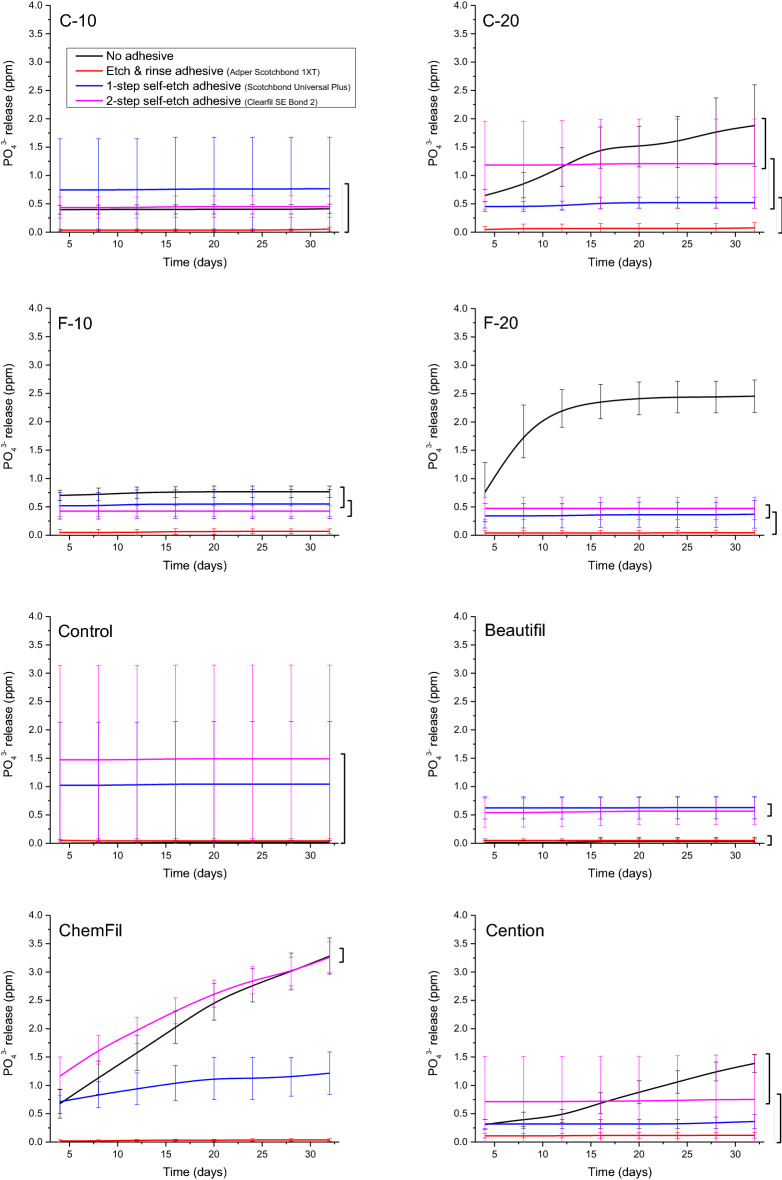
Cumulative concentrations of phosphate ions measured in lactic acid solution over 32 days. Square brackets indicate statistically similar values at the end of the observation period.

Figure 3 shows that PO_4_ concentrations for most of the materials (C-20, F-10, F-20, ChemFil, Cention) were significantly influenced by the adhesive type with the following rankings: uncoated ≥ 2-SEA ≥ 1-SEA ≥ ERA. Beautifil was regarded as a non-PO_4_-releasing material according to zero release from uncoated specimens, however, a statistically significant PO_4_-release from Beautifil was identified for specimens coated with 1-SEA and 2-SEA. No significant effect of the adhesive coating on PO_4_ concentrations was identified for C-10 and Control due to high data variability and consequently small effect size.

**Figure 4:**
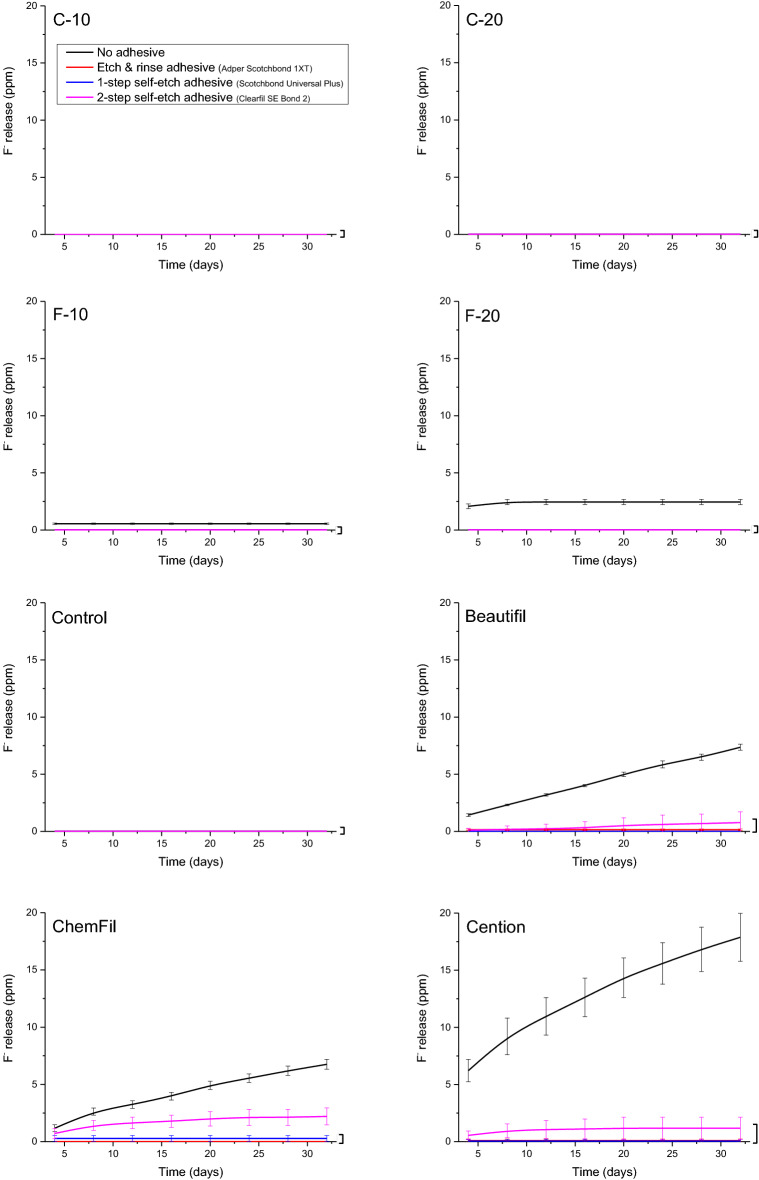
Cumulative concentrations of fluoride ions measured in lactic acid solution over 32 days. Square brackets indicate statistically similar values at the end of the observation period.

For all of the F-releasing materials (F-10, F-20, Beautifil, ChemFil, Cention), the uncoated specimens showed significantly higher F concentrations than the adhesive-coated specimens (Fig. 4). The F concentrations measured for different adhesive coatings were statistically similar, except for ChemFil, which showed significantly higher F concentrations for 2-SEA compared to 1-SEA and ERA.

**Figure 5:**
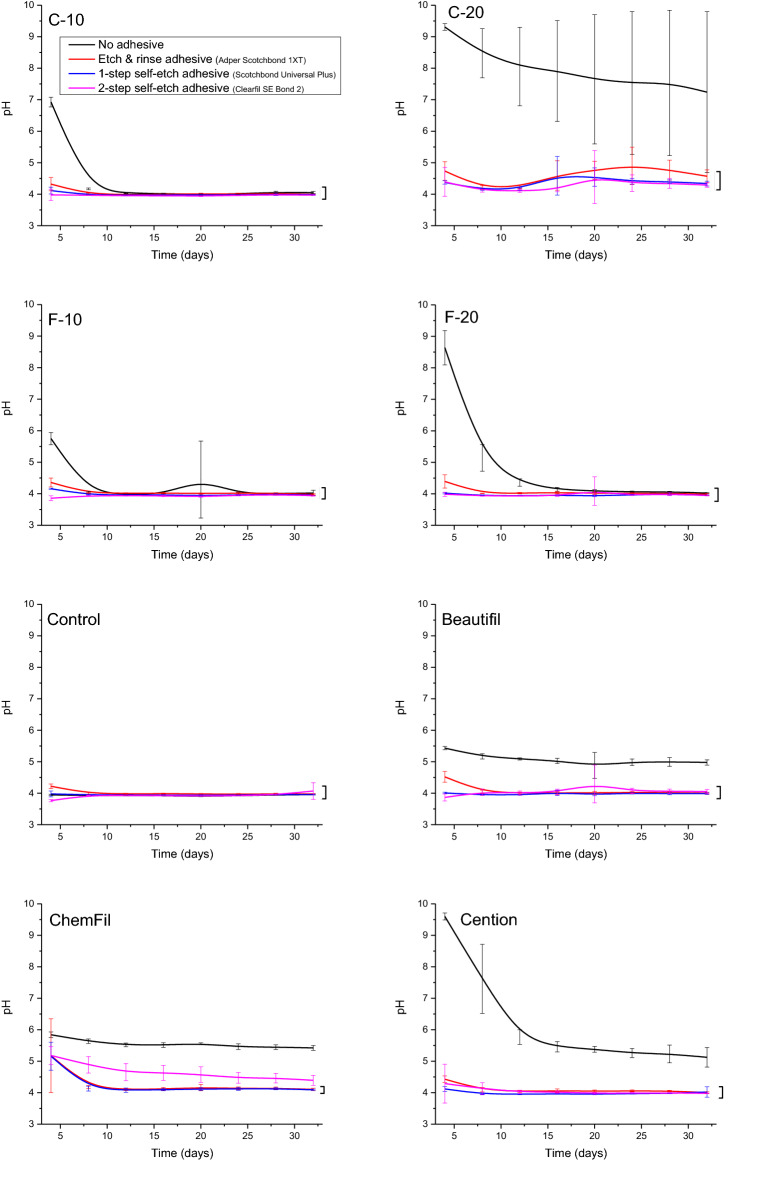
pH changes of immersion solution over 32 days. Square brackets indicate statistically similar values at the end of the observation period.

Among the BG-containing composites, only C-20 maintained a significantly higher pH throughout the whole observation period, while for the other BG-containing composites pH increase was transient and lasted for 4–16 days (Fig. 5). The commercial materials showed comparatively longer-lasting pH release with the final pH values for uncoated specimens of 5.0–5.4. Considering the final pH, lower values were observed for the adhesive-coated specimens compared to uncoated specimens for all commercial materials, as well as for the only experimental material capable of long-lasting alkalization (C-20). There were no significant differences in the final pH values among different adhesive types, except for ChemFil in which 2-SEA led to a higher pH compared to 1-SEA and ERA.

**Figure 6:**
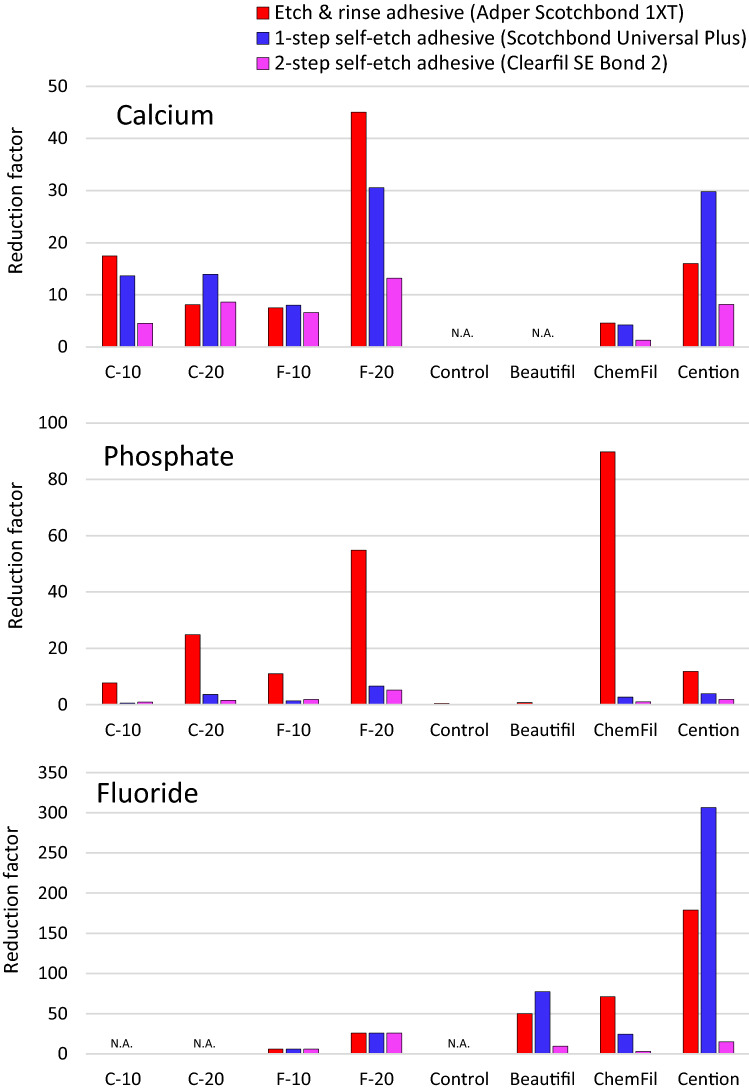
Reduction factors for calcium, phosphate, and fluoride ions calculated by diving the concentrations of uncoated specimens by the corresponding concentrations of adhesive-coated specimens.

Figure 6 shows the reduction factors calculated by dividing cumulative 32 days ion concentrations of uncoated specimens by the corresponding concentrations measured for adhesive-coated specimens. The reduction factors varied for three orders of magnitude (0.3–306.5) reflecting extreme differences depending on ion types, restorative materials, and adhesives. F release was most highly impaired, with a reduction factor of up to 307, followed by PO_4_ and Ca with maximum reduction factors of 90 and 45, respectively.

**Figure 7:**
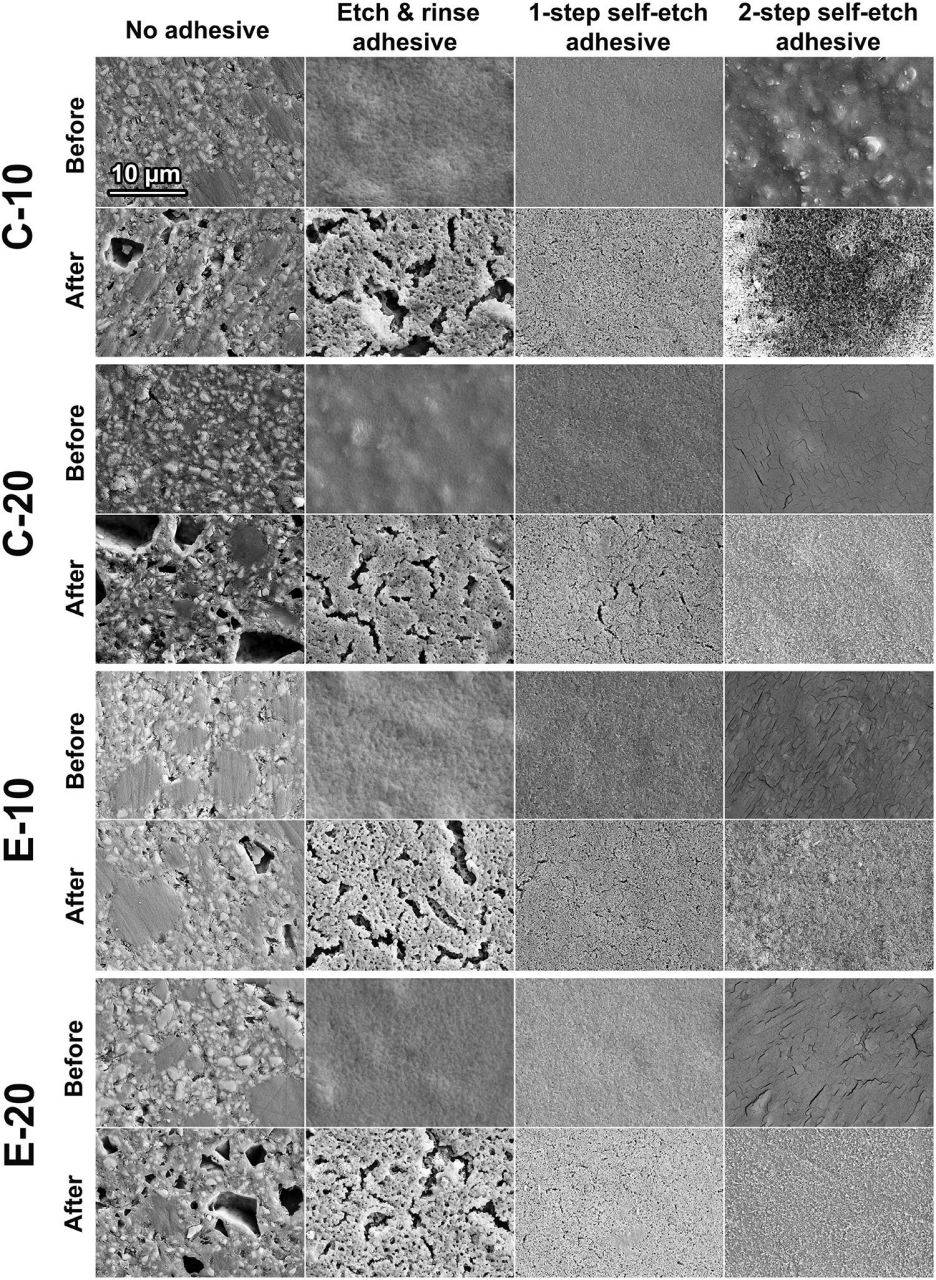
Scanning electron micrographs of bioactive glass-containing experimental composites before and after 32 days lactic acid immersion.

**Figure 8:**
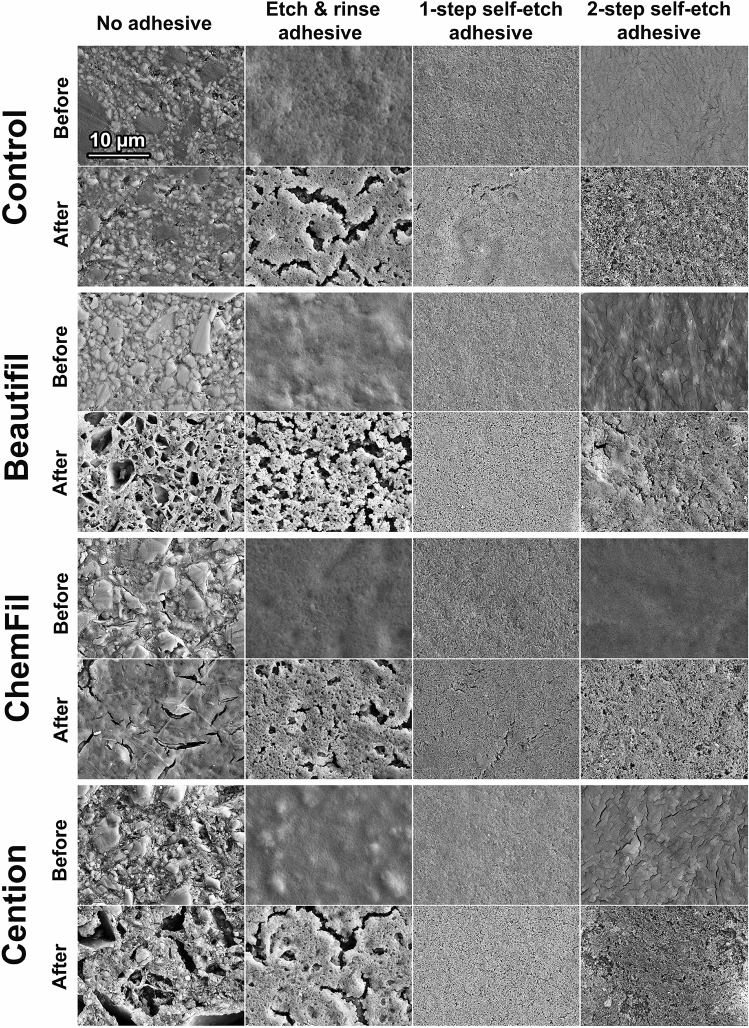
Scanning electron micrographs of the control composite and commercial restorative materials before and after 32 days lactic acid immersion.

SEM images of specimen surfaces before and after the 32 days lactic acid immersion are shown in Figs. 7 and 8. Uncoated specimen surfaces before immersion showed irregular filler particles of various sizes surrounded by the resin matrix (for the composites) or polysalt matrix (for the glass ionomer). After the 32 days immersion, filler degradation was evidenced by irregular pits that correspond to the size and shape of filler particles. SEM images of adhesive-coated specimen surfaces showed comparatively less variability among the materials and had a more consistent appearance within a given adhesive system. Before immersion, the adhesive-coated surfaces showed no distinct surface characteristics, as the surface morphology of restorative materials was hidden by a smooth layer of adhesive resin. After immersion, the adhesive-coated surfaces showed signs of degradation due to the swelling and dissolution, followed by desiccation as part of specimen preparation for SEM analysis. These post-immersion defects in the adhesive surface were most pronounced for ERA, whereas the other two adhesives showed a comparatively more homogeneous layer with fewer cracks.

**Figure 9:**
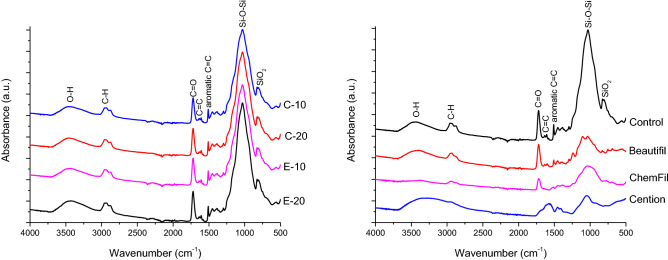
Representative Fourier-transform infrared spectra of uncoated restorative material surface before 32 days lactic acid immersion.

**Figure 10:**
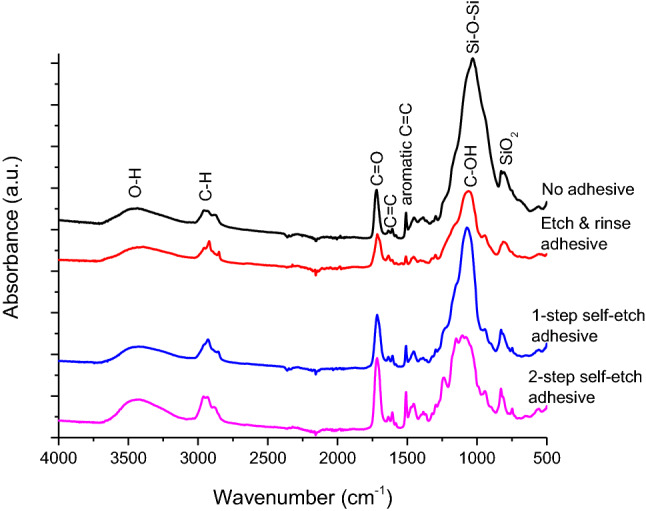
Representative Fourier-transform infrared spectra of adhesive-coated surface of the experimental composite E-20 before 32 days lactic acid immersion.

Representative FTIR spectra collected from surfaces of uncoated specimens of restorative materials before lactic acid immersion are shown in Fig. 9. The experimental and commercial composite materials presented spectral features typical for glass/silica-filled methacrylate-based materials, while the spectrum for ChemFil presented broad peaks typical for glass ionomers. Since FTIR spectra of adhesive-coated specimens practically reflected the composition of the overlying adhesive layer, without significant spectral contributions from underlying restorative materials, Fig. 10 shows spectra of adhesive-coated specimens for only one representative material (E-20).

**Figure 11:**
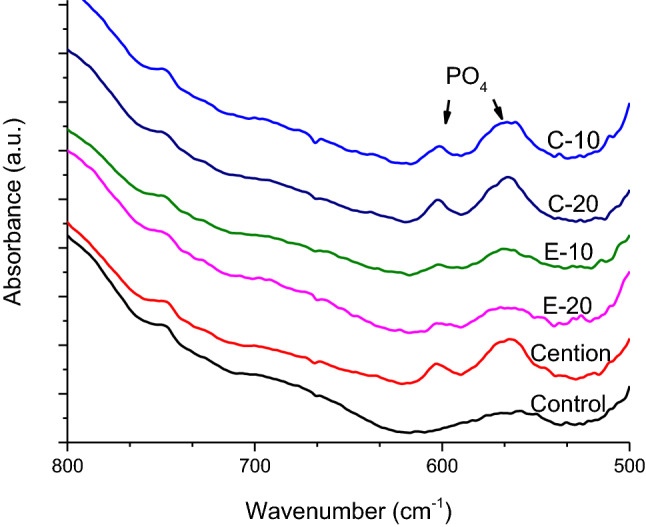
Representative Fourier-transform infrared spectra of specimen surface on which hydroxyapatite precipitate was identified after 32 days lactic acid immersion. The spectral bands at 560 and 600 cm^−1^ were assigned to bending mode of the phosphate group.

The part of the FTIR spectra used for the identification of hydroxyapatite precipitate after the 32 days lactic acid immersion is shown in Fig. 11. This figure shows spectra for the experimental groups in which hydroxyapatite was present (C-10, C-20, E-10, E-20, and Cention), and the control material. The spectra of other experimental groups are omitted, as these showed no spectral bands corresponding to PO_4_ vibrations.

## Discussion

This in vitro study builds on a series of previous studies that investigated experimental BG-functionalized composites regarding their acid-neutralizing effects^32^, shrinkage properties^33^, ion release^23^, and anti-demineralizing protective effects on enamel^21^ and dentin^22^. The present study addressed the potential of adhesive coating for hindering the reactivity of the experimental BG-containing composites and selected commercial ion-releasing restorative materials. The results showed that three representative contemporary adhesives reduced ion release between 0.3 and 307 times, in a significantly material- and adhesive-dependent manner, leading to the rejection of the null hypothesis.

The ion-releasing capability of composites functionalized with reactive fillers depends on their permeability to water, as sufficient water absorption is necessary for ions to be released from the fillers and leached into the surrounding solution. The overall permeability of a composite material is determined by the level of hydrophilicity of its resins and fillers. Highly hydrophilic resin systems containing hydroxyethyl methacrylate (HEMA) were deliberately used in earlier studies to improve ion release from experimental composites^34^, as “conventional” hydrophobic resin mixtures hindered the material’s reactivity^35^. However, the resin system does not necessarily need to be highly hydrophilic by design as the water-soluble unsilanized reactive fillers themselves make the composite structure more porous and water-permeable^36^. Hence, the experimental composites in the present study were prepared using a more hydrophobic resin system (Bis-GMA/TEGDMA) to which hydrophilic BG-fillers were added, leading to sufficient hydrophilicity for ion release and remineralizing effects^21–23^ without excessively sacrificing mechanical properties^37^. Unlike the experimental composites, which can be tailored for appropriate hydrophilicity, adhesive systems should create a layer as hydrophobic as possible in order to resist hydrolytic and enzymatic degradation^38^. The hydrophobic adhesive layer works against water diffusion through the material, hindering the release of remineralizing ions. In addition to acting as a chemical-physical barrier for ion release, self-etch adhesives may additionally diminish Ca release from remineralizing materials via its binding by acidic monomers as carboxylate salts^20^.

Notwithstanding the facts that a wide range of experimental remineralizing composites have been exhaustively investigated^1,8,34,39–44^ and that some remineralizing composite materials such as giomers and alkasite are being successfully used in clinical practice, the negative influence of the adhesive layer on ion release has rarely been considered. The effect of the adhesive appears unimportant if only the outer restoration surface is expected to release remineralizing ions. However, the influence of the adhesive layer becomes relevant if potential benefits of remineralizing restorative materials at restoration sites adjacent to cavity walls are considered. As the tooth/restoration interface of composite restorations is in reality never perfectly intact^45^, the susceptibility of a locally failed margin for the spreading of secondary caries could be reduced if reactive restorative materials could also exert their remineralizing effects at this site. However, according to the presented results, the adhesive-coated part of the restoration is much less reactive, implying that the protective effects of ion-releasing materials are mostly limited on preventing secondary caries at the outermost site of the dentin/restoration interface, i.e., at cavosurface margins. Once the initial lesion advances along the marginal gap^46^, the material’s remineralizing effects can be expected to diminish considerably.

Despite being limited to fluoride ions, previous studies have quantified the negative effect of the hydrophobic resinous layer on ion release and reported that resin-coated glass ionomer cements released 45–78% less fluoride than uncoated samples, while for fluoride-releasing composites, adhesive coating reduced fluoride release for 91–96%^13^. Other studies reported that adhesive coating reduced fluoride release up to 30 times for alkasite, giomer, and glass ionomer^19^, 2–3 times for polyacid-modified composites^16^, whereas for a commercial F-containing composite, fluoride release was diminished to undetectable levels^18^. All of the aforementioned studies agree in their principal conclusions that the reduction of fluoride release caused by a resinous coating may impair the material’s potential to prevent secondary caries. Although the findings of the present study cannot be quantitatively compared to the other studies due to considerably different methodologies and investigated materials, the general conclusion that the adhesive coating impairs ion release from restorative materials to widely different extents is in accordance with previous studies.

Different types of adhesives are characterized by different hydrophilicity, which is dictated by their mode of application. Generally, adhesives which feature a separate application of an additional “second bottle” component tend to be more hydrophobic than “one-bottle” adhesives^38,47^. In this regard, 2-SEA in the present study was expected to create a less permeable barrier for ion release than the other two adhesives, which do not contain an additional hydrophobic “second bottle” component (ERA and 1-SEA). It was thus surprising to observe the opposite results, i.e., that 2-SEA allowed generally higher ion release than 1-SEA and ERA. Moreover, there was no particular pattern or ranking of the investigated adhesives regarding their potential for diminishing ion release, except for the finding that ERA reduced PO_4_ release more than the other two adhesives in all PO_4_-releasing materials. Overall, by investigating three representative adhesive systems, the present study identified no patterns that would be specific for particular adhesive classes, i.e., the results were more dependent on the individual brand of adhesive than on its classification into a particular group. Despite belonging to different “generations” and different modes of application, all three investigated adhesives contain HEMA, while the solvent was water (for 2-SEA) or ethanol/water (for ERA and 1-SEA). This compositional information provided by the manufacturers is only qualitative and thus insufficient for making mode detailed inferences about relative hydrophilicities of the tested adhesives. Without quantitative compositional information made available by the manufacturers, the commercial products can only be investigated as integrated complex systems, hence no attempts were made in the present study to discern the contributions of their individual components.

The fact that specimen surfaces were homogeneously covered with an adhesive coating that diminished ion release was evidenced by SEM images (showing disappearance of characteristic filler/matrix micromorphology in adhesive-coated specimens) and FTIR analysis (showing that spectral fingerprint of adhesives dominated the spectra of material surface, with no observable contribution of underlying restorative material). Although SEM images indicated signs of degradation and fracturing of the adhesive layer after the 32 days lactic acid immersion, it cannot be differentiated whether these processes occurred during the lactic acid immersion or later due to specimen desiccation. In accordance with the findings of a previous study on ion release from uncoated restorative materials^23^, the experimental BG-containing composites and the commercial material Cention showed the capability for hydroxyapatite precipitation even in the acidic solution. It appears that this precipitation can occur if local pH values near the specimen surface are sufficiently elevated, without the need for alkalization of the whole volume of the immersion solution. The lack of hydroxyapatite precipitation in the adhesive-coated experimental groups is aligned with their diminished ion release and the fact that filler particles that served as nucleation spots were insulated from the aqueous solution by a resinous layer.

In studies of the effect of resinous coating on ion release from glass ionomer cements, specimens were commonly coated with corresponding products recommended by their respective manufacturers^14,19^. In the present study, all restorative materials (including the glass ionomer) were coated using adhesive systems intended for use with resin composites. Coating the glass ionomer with adhesive systems was done to ensure a full factorial design for all materials (3 adhesives × 8 materials) and is clinically relevant as a glass ionomer/adhesive system interface commonly occurs between the glass ionomer restoration base and the composite part of the restoration. In such a laminated restoration, the glass ionomer base is usually covered with an adhesive layer on which the composite overlay is placed. Should the dentin/composite interface fail and the leakage extend to the glass ionomer base, its ion release through the adhesive layer would be beneficial for preventing further demineralization.

The experimental approach based on immersion of restorative material specimens in lactic acid solution (pH = 4.0) was used according to a previous study^23^, in which it proved convenient for measuring concentrations of individual ions under moderately acidic conditions, which accelerated the dissolution of reactive fillers. Neutral solutions or solutions of higher ionic strengths (e.g., artificial saliva) would reduce ion release, making the differences among materials less pronounced and for some experimental groups even diminishing ion concentrations to undetectable levels. Although the present study, as well as other in vitro studies on the topic^13–20^ are unable to account for complex processes occurring in the clinical environment, including variations in saliva flow rates, ionic activities of the immersion solution, and cycles of acid production, the simple experimental setup enabled identifying highly pronounced and material-dependent effects of the adhesive coating on the reactivity of both the experimental BG-functionalized and the commercial ion-releasing restorative materials.

A detailed qualitative and quantitative analysis of ion release from the experimental and commercial materials investigated in the present study were reported in a previous article^23^, while their protective effects were also addressed previously^21,22^. Hence, these aspects were not discussed in detail in the present work, which aimed to highlight the effect of adhesive coating on ion release. An additional note should be made regarding the fact that for the non-releasing composite (Control), positive PO_4_ values were measured for 1-SEA and 2-SEA. Although the malachite green assay used in the present study to quantify PO_4_ measured only free phosphate in the solution, the self-etch adhesives contain phosphate groups in acidic monomers which can be liberated by hydrolysis, leading to non-zero PO_4_ concentrations for the control composite that was otherwise not expected to release any ions.

Regarding the relative importance of calcium and phosphate ions for remineralization of dental hard tissues, calcium release from remineralizing materials is comparatively more efficient than phosphate release. Since a calcium/phosphate ratio of 1.6 is considered optimal for remineralization and dental plaque contains an excess of phosphate ions (calcium/phosphate ratio of 0.3^48^), calcium has been regarded as the main remineralization rate-limiting component. Hence, small changes in calcium concentration exert disproportionately better remineralizing effects than comparable changes in phosphate concentrations^49^. In quantitative terms, calcium was shown to be approximately 20 times more effective than phosphate in preventing enamel demineralization, i.e., for a similar reduction in demineralization, 20 times higher phosphate concentrations were required compared to calcium concentration^50^. Also, considering the stoichiometry of hydroxyapatite, phosphate concentration contributes to the ionic activity product much less than calcium concentration as the concentrations of these ions are raised to the third and fifth power, respectively^50^. Despite being already present in saliva in sufficient concentrations and comparatively less important than calcium, phosphate ions released from restorative materials can nevertheless contribute to inhibition of acid-induced demineralization of dental hard tissues, albeit to a lesser extent^51^.

Cention is a recently launched commercial ion-releasing restorative material which is similar to its manually-mixed predecessor Cention N. Due to the compositional complexity of these materials and incomplete information about their proprietary ingredients, the high calcium and fluoride ion release observed in the present study cannot be decisively attributed to any of Cention’s particular features; however, the overall material behaviour can be discussed in the context of previous studies. Cention/Cention N is based on a methacrylate resin filled with conventional barium alumino-silicate glass as inert reinforcing filler, and two reactive glasses, namely a calcium barium alumino-fluoro-silicate ionomer glass, and a basic calcium fluoro-silicate glass^4^. The latter glass undergoes a degradation process similar to that of other bioactive glasses that have been investigated as prospective fillers in ion-releasing restorative composites. The main difference between these “conventional” bioactive glasses and the reactive glass in Cention is that the latter is phosphate-free. To demonstrate the capability of this glass to precipitate apatite and release calcium and fluoride ions, a compositionally similar model SiO_2_–CaO–CaF_2_–Na_2_O glass was synthesized and characterized in a recent study^52^. That glass had a network connectivity of 2.36 and was expectedly less reactive than the traditional 45S5 composition. However, the ion-releasing behaviour of Cention is additionally affected by the presence of ionomer glass and ytterbium trifluoride, partially explaining the high ion release but also rendering the contributions of individual components to the overall ion release profile difficult to separate. In the context of high ion release from Cention, it should be mentioned that a recent study on eleven contemporary ion-releasing restorative materials indicated a very high potential of Cention N for calcium release in lactic acid solution, as well as re-release following fluoride recharge^24^. In that study, Cention’s calcium release ranked the highest among all of the tested materials, including high-viscosity glass ionomer cements, resin-modified glass ionomer cements, and other resin-based ion releasing materials (e.g., giomers). The potential clinical benefits of high ion release from Cention N are substantiated by the report of its capability to remineralize artificial interproximal enamel caries^53^.

Some commercial and experimental ion-releasing restorative materials are known to precipitate hydroxyapatite on their surface^7,9,23,32^. The precipitation starts with heterogeneous nucleation on filler particles, after which hydroxyapatite crystals grow by incorporation of ions available in the surrounding solution, as well as these leached from the material itself. BGs are known to facilitate the process of heterogeneous nucleation as their dissolution involves the formation of hydrated silica gel which acts as a nucleation site^54^. Even phosphate-free BGs are capable of precipitating hydroxyapatite given that a sufficient amount of phosphate is available in the solution. This is the case for Cention, which contains a phosphate-free BG, but its ionomer glass serves as a source of phosphate ions when exposed to acidic pH values^52^. Under low-pH conditions at the start of each 4 days acid immersion cycle, phosphate is released, whereas the subsequent pH increase (occurring at least locally at the surface of BG particles) favors hydroxyapatite precipitation. In addition to Cention, surface formation of hydroxyapatite also occurred in the uncoated BG-containing experimental composites, as indicated by their FTIR spectra. The mechanism of heterogeneous nucleation is the same as for Cention, with the difference of phosphate ions originating from BG (as both 45S5 and the customized BG contain phosphate) instead of the ionomer glass. The surface precipitation of hydroxyapatite or fluorapatite could be used to seal marginal gaps at the sites of failed adhesion between the adhesive and the adjacent cavity wall^7^.

Although ion release from restorative dental materials is commonly regarded as a potential means for protection against secondary caries, it should be noted that strong clinical evidence for this claim still does not exist. The main argument against effective protection from secondary caries is based on the limited time availability of ion release from restorative materials. Other potential benefits of ion-release from restorative materials which are less affected by its short duration are remineralization of caries-affected dentin and promoting mineral precipitation within the hybrid layer. Both of these effects can be impaired by the interposition of an adhesive coating between the ion-releasing material and the substrate to be remineralized.

## Conclusion

The known antagonism between the ion-releasing capability and stability of remineralizing restorative materials should also be observed if these materials are expected to release ions through the adhesive interface. Whereas a hydrophobic character of adhesive systems is preferable for maintaining hybrid layer stability, the resinous adhesive layer can act as a barrier for ion release and diminish the beneficial effects of remineralizing restorative materials. The present study demonstrated that for both experimental and commercial remineralizing restorative materials the release of calcium, phosphate, and fluoride ions from their adhesive-coated surface (mimicking the conditions at the tooth/restoration interface) can be up to three orders of magnitude lower compared to the outer restoration surface that remains uncoated.

## Data Availability

The datasets generated during and/or analysed during the current study are available from the corresponding author on reasonable request.
